# Supplementary material for: Pressure-enhanced f-electron orbital weighting in UTe2 mapped by quantum interferometry
Source: Commun Phys. 2025 Nov 19;8(1):454. doi: 10.1038/s42005-025-02333-5 (PMC12629984; doi:10.1038/s42005-025-02333-5)
Supplement: Supplementary file 1 — Supplementary Information [file 42005_2025_2333_MOESM1_ESM.pdf]

Supplementary information for:

Pressure-enhanced f-electron orbital weighting in UTe_2 mapped by quantum interferometry

T. I. Weinberger, Z. Wu, A. J. Hickey, D. E. Graf, G. Li, P. Wang, R. Zhou, A. Cabala, J. Pu, V. Sechovský, M. Vališka, G. G. Lonzarich, F. M. Grosche, A. G. Eaton

Correspondence to: alex.eaton@phy.cam.ac.uk

Supplementary Note 1 – Relationship between Quantum Interference Oscillation Frequencies and Fermi Surface Warping

The quantum interference oscillation (QIO) frequency observed for magnetic field oriented parallel to the a-axis of UTe_2 can be related to the degree of warping of the electron pocket of the Fermi surface in our parameterized Fermi surface model.[1] The 220 T QIO arises from quasiparticles moving along k_z on the electron and hole sheets (see Fig.1e in the main text). These oscillations correspond to the warping of the electron cylinder in the k_y direction as it deviates away from, and returns towards, the hole cylinder. While the hole cylinder follows a straight vertical trajectory in k_z when viewed along the a-axis, the electron cylinder traces a sinusoidal path in k_y, described by

$$d_y = r_e\left(1 - \cos(k_z)\right), \tag{1}$$

where r_e is the warping amplitude. In our ambient pressure Fermi surface model, we determined this warping parameter to be $0.006a_0^{-1}$ (with a_0 as the Bohr radius), based on fits to our

Fermi surface model.[1,2] The area of the resulting loop is given by

$$A = \int_{-\pi}^{\pi} r_e \left(1 - \cos(k_z)\right) dk_z = 2\pi r_e. \tag{2}$$

Our parameterised model yields an enclosed area corresponding to a QIO frequency of 207 T at 0 kbar, in close agreement to the measured value of 220 T.

Supplementary Note 2 – Probability Dependence of Quantum Interference Oscillations and Harmonics

As discussed in the main text, the probability dependence of the first harmonic of a QIO for fields along the a-axis is $P_{QIO_1} \propto P^2Q^2$ where P is the probability of a breakdown event and $Q = 1 - P$ is the probability of avoiding a breakdown event. To observe the second harmonic, two further breakdown events must be avoided such that $P_{QIO_2} \propto P^2Q^4$.

This gives rise to the interesting property that although as $P \rightarrow 0$ (i.e. there is no breakdown) both $P_{QIO_1} \rightarrow 0$ and $P_{QIO_2} \rightarrow 0$, the relative amplitude of the second harmonic to the first goes $P_{QIO_2}/P_{QIO_1} \rightarrow 1$ (Fig. S1a). While a full treatment would also consider higher harmonics, for simplicity here we shall restrict our considerations to the first two. The breakdown probability depends on the breakdown field B_0:

$$P = \exp(-B_0/B\cos\theta). \tag{3}$$

The breakdown field is proportional to the square of the k-space gap between Fermi surface sheets, $B_0 \propto k_g^2$ (ref.[3]). As k_g increases, P_{QIO_2}/P_{QIO_1} approaches 1. We observe that as we increase the pressure under which our UTe_2 sample is subjected, the ratio of the second harmonic to the first harmonic increases, which we interpret as indicating that the gap between the Fermi surface sheets grows (Fig. S1b).

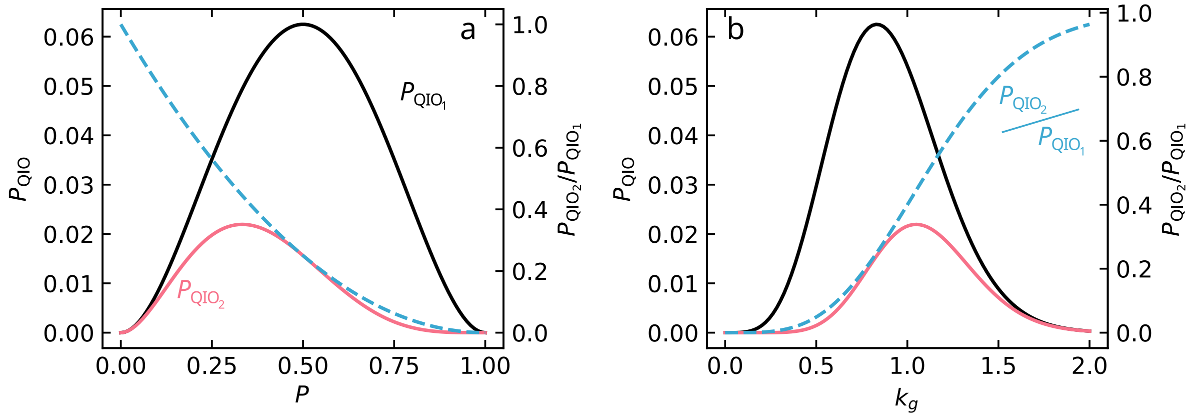

Fig. S1. (a) Probability of the first harmonic QIO (black) and the second harmonic QIO (red) occurring as a function of breakdown probability P (indicative values from the left-hand axis). Since both breakdown events and avoided breakdown events are required for a QIO to occur, the QIO probability does not monotonically increase with increased breakdown probability. Instead, it exhibits a well-defined peak in the range $P \in [0, 1]$. Interestingly, the ratio of the second harmonic to the first harmonic, P_{QIO_2}/P_{QIO_1} (dashed blue), increases as the probability of breakdown decreases (indicative value on the right-hand axis). (b) P can be related to the k-space gap between Fermi surface sheets by the functional form $P \approx \exp(-k_g^2)$. This shows that as $k_g \to \infty$, $P_{QIO_2}/P_{QIO_1} \to 1$. Our data show that P_{QIO_2}/P_{QIO_1} grows under pressure. Consequently, we interpret that as indicating the gap between Fermi sheets grows as pressure is increased.

Supplementary Note 3 – Comparison of Tight-Binding and GGA+U Band Structures

To investigate the orbital contributions at the Fermi level, we developed a minimal six-orbital tight-binding model. Since UTe_2 adopts an inversion-symmetric *Immm* crystal structure and is paramagnetic at ambient pressure and zero magnetic field, spin polarisation is neglected. The model assumes that the Fermi surface is primarily influenced by U f- and d-orbitals and Te p-orbitals. The unit cell contains two uranium atoms (each contributing f- and d-orbitals) and four tellurium atoms (with only the Te-2 atoms contributing p-orbitals at the Fermi level). This f-d-p model is consistent with previous studies.[4,5] Initial hopping parameters were based on Ishizuka *et al.*[4] and iterated to match our quantum oscillation data (Figure S2).

The geometry of the Fermi surface is insensitive to the absolute values of the hopping parameters, provided they are all scaled equally. Therefore, to confirm the validity of our model and rescale the parameters, we compared our tight-binding model to a density functional theory (DFT) calculation for UTe_2 with internal coordinates shifted to produce an improved fit to our quantum oscillation data (Figure 4).

We performed DFT calculations for UTe_2 using the full-electron, linearised augmented plane-wave package Wien2K.[6] The electronic structures were converged on a $17 \times 17 \times 17$ Monkhorst-Pack k-mesh within the Brillouin zone of the primitive unit cell using the Generalized Gradient Approximation exchange-correlation potential. A Hubbard parameter, U, of 8 eV was applied, with the static magnetic moment on uranium ions constrained to zero. Spin-orbit coupling effects were also included. Lattice parameters and atomic positions were initialised based on prior DFT studies[7,8] and then perturbed to better fit quantum oscillation data of UTe_2 (Table S1).

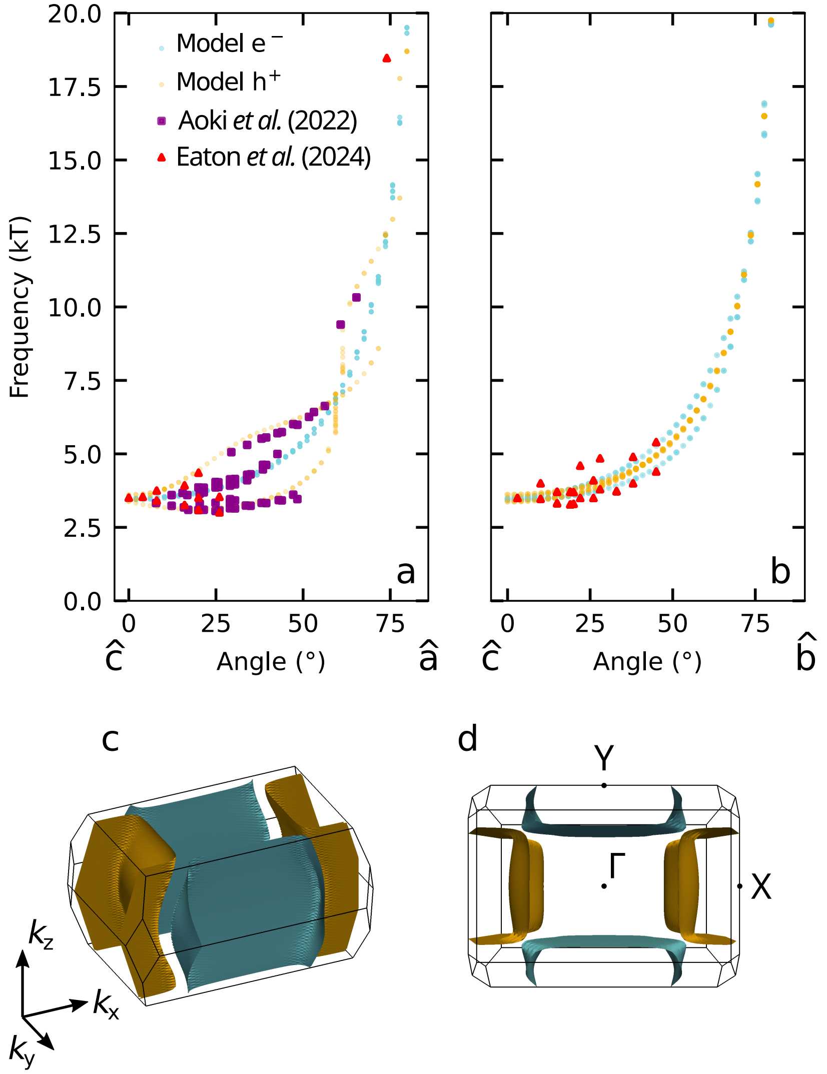

Fig. S2. The Fermi surface of the tight-binding model and its fit to quantum oscillation data are shown for (a) the c-a magnetic field tilt plane and (b) the c-b plane.[1,8] Pronounced warping of the hole sheet is evident in (c) the side view of the Fermi surface, showing the undulating warping of the Fermi sheets, also visible from (d) the top-down view.

Species	x	y	z
U	0	0	0.13473
Te1	0.5	0	0.26299
Te2	0	0.21438	0.5

Table S1: The internal atomic positions used in our DFT study of UTe_2. The lattice constants chosen for the calculations were a = 4.16 Å, b = 6.13 Å, and c = 13.96 Å.

Supplementary Note 4 – Orbital Weighting of f-, d-, and p-orbitals

The orbital weighting of the tight-binding model's bands and Fermi surface highlights the relationship between Fermi surface geometry and orbital character. The two bands with the strongest f-orbital character exhibit narrow bandwidths, consistent with heavy, localized f-electron behaviour. These f-bands do not cross the Fermi level, resulting in no additional 3D pockets at the Γ or Z points. However, significant f-d hybridisation in the d-dominated bands (Figure 4) explains the heavy quasiparticle masses observed by quantum oscillation measurements and the f-orbitals' influence on electronic and superconducting properties, even without forming Fermi sheets.

The d-bands, split around the Fermi level, have wider bandwidths and lower effective masses compared to f-bands, with the lower d-band crossing the Fermi level. In contrast, p-bands are also split about the Fermi level, with the upper band contributing to an electron-like Fermi surface sheet. The p-bands show limited f- and d-character except between the Γ and R high-symmetry points.

The tight-binding model accurately reproduces the Fermi surface's orbital contributions (Figure S3). Fermi surface sheets perpendicular to the k_y-direction are primarily p-dominated, while those perpendicular to the k_x-direction are d-dominated. The f-character is most prominent on the warped face of the hole sheet perpendicular to k_x-direction, aligning with the quasi-2D behaviour of the Fermi surface and suggesting that f-electrons drive this effect. This could have important consequences for the nature of spin fluctuations in UTe_2.

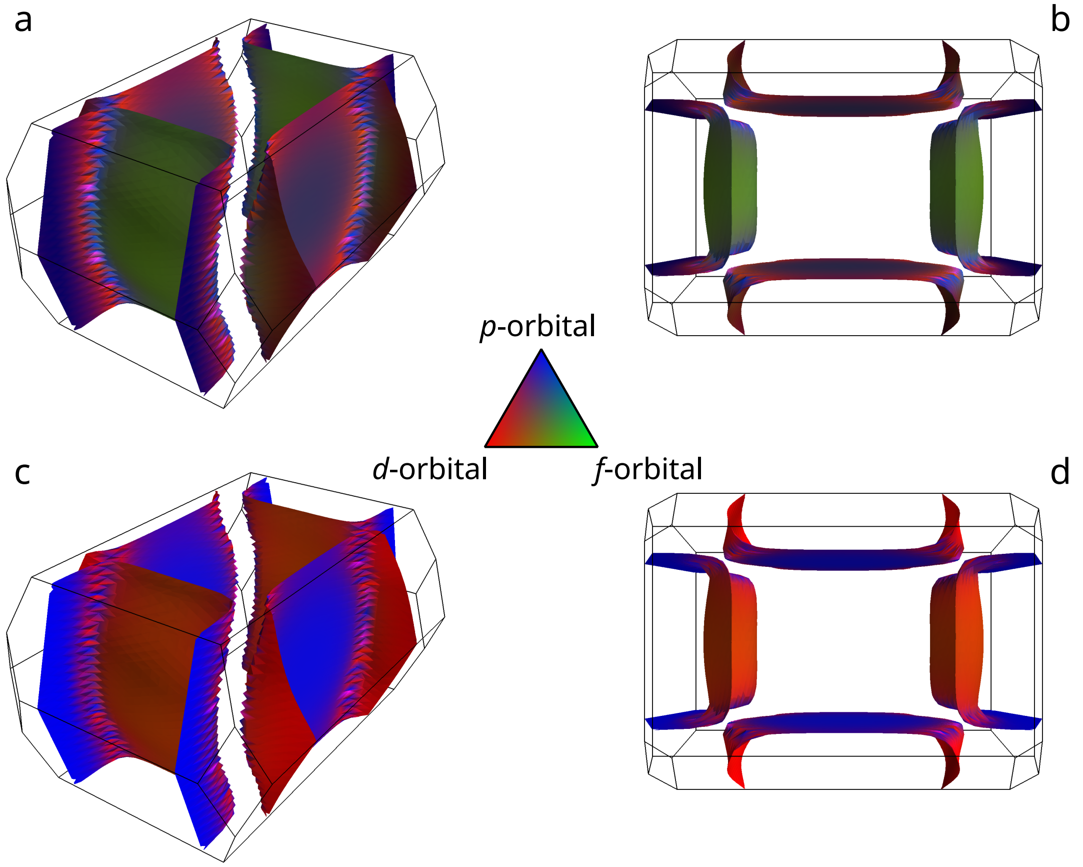

Fig. S3. (a, b) Orbital contributions of p- (blue), d- (red), and f-orbitals (green) to the Fermi surface sheets. The f-orbitals predominantly contribute to the warped face of the hole cylinder oriented perpendicular to the k_x-direction. (c, d) The squared orbital contributions reveal that the Fermi surface is primarily of p- and d-character, with p-orbitals dominating the character of FS sheets perpendicular to the k_y-direction and d-orbitals perpendicular to the k_x-direction.

Supplementary References

1. Eaton, A. G. *et al.* Quasi-2D Fermi surface in the anomalous superconductor UTe_2. *Nat. Commun.* **15**, 223 (2024).

2. Weinberger, T. I. *et al.* Quantum Interference between Quasi-2D Fermi Surface Sheets in UTe_2. *Phys. Rev. Lett.* **132**, 266503 (2024).

3. Chambers, R. G. Magnetic breakdown in real metals. *Proc. Phys. Soc.* **88**, 701 (1966).

4. Ishizuka, J. & Yanase, Y. Periodic Anderson model for magnetism and superconductivity in UTe_2. *Phys. Rev. B* **103**, 094504 (2021).

5. Haruna, S., Nomura, T. & Kaneyasu, H. Possible Unconventional s-Wave Pairing with Point-Node–Like Gap Structure in UTe_2. *J. Phys. Soc. Jpn.* **93**, 1347–4073 (2024).

6. Blaha, P. *et al.* WIEN2k: An APW+lo program for calculating the properties of solids. *J. Chem. Phys.* **152**, 074101 (2020).

7. Ishizuka, J., Sumita, S., Daido, A. & Yanase, Y. Insulator-Metal Transition and Topological Superconductivity in UTe_2 from a First-Principles Calculation. *Phys. Rev. Lett.* **123**, 217001 (2019).

8. Aoki, D. *et al.* First Observation of the de Haas–van Alphen Effect and Fermi Surfaces in the Unconventional Superconductor UTe_2. *J. Phys. Soc. Jpn.* **91**, 083704 (2022).
